# Gadolinium Doping Modulates the Enzyme-like Activity and Radical-Scavenging Properties of CeO_2_ Nanoparticles

**DOI:** 10.3390/nano14090769

**Published:** 2024-04-26

**Authors:** Madina M. Sozarukova, Taisiya O. Kozlova, Tatiana S. Beshkareva, Anton L. Popov, Danil D. Kolmanovich, Darya A. Vinnik, Olga S. Ivanova, Alexey V. Lukashin, Alexander E. Baranchikov, Vladimir K. Ivanov

**Affiliations:** 1 Kurnakov Institute of General and Inorganic Chemistry of the Russian Academy of Sciences, 119991 Moscow, Russia; 2 Materials Science Department, Lomonosov Moscow State University, 119234 Moscow, Russia; 3 Institute of Theoretical and Experimental Biophysics of the Russian Academy of Sciences, 142290 Pushchino, Russia; 4 Frumkin Institute of Physical Chemistry and Electrochemistry of the Russian Academy of Sciences, 119071 Moscow, Russia

**Keywords:** nanozyme, nanoceria, gadolinium, conjugate, antioxidant, prooxidant, radical scavenger, chemiluminescence

## Abstract

Their unique physicochemical properties and multi-enzymatic activity make CeO_2_ nanoparticles (CeO_2_ NPs) the most promising active component of the next generation of theranostic drugs. When doped with gadolinium ions, CeO_2_ NPs constitute a new type of contrast agent for magnetic resonance imaging, possessing improved biocatalytic properties and a high level of biocompatibility. The present study is focused on an in-depth analysis of the enzyme-like properties of gadolinium-doped CeO_2_ NPs (CeO_2_:Gd NPs) and their antioxidant activity against superoxide anion radicals, hydrogen peroxide, and alkylperoxyl radicals. Using an anion-exchange method, CeO_2_:Gd NPs (~5 nm) with various Gd-doping levels (10 mol.% or 20 mol.%) were synthesized. The radical-scavenging properties and biomimetic activities (namely SOD- and peroxidase-like activities) of CeO_2_:Gd NPs were assessed using a chemiluminescent method with selective chemical probes: luminol, lucigenin, and L-012 (a highly sensitive luminol analogue). In particular, gadolinium doping has been shown to enhance the radical-scavenging properties of CeO_2_ NPs. Unexpectedly, both bare CeO_2_ NPs and CeO_2_:Gd NPs did not exhibit SOD-like activity, acting as pro-oxidants and contributing to the generation of reactive oxygen species. Gadolinium doping caused an increase in the pro-oxidant properties of nanoscale CeO_2_. At the same time, CeO_2_:Gd NPs did not significantly inhibit the intrinsic activity of the natural enzyme superoxide dismutase, and CeO_2_:Gd NPs conjugated with SOD demonstrated SOD-like activity. In contrast to SOD-like properties, peroxidase-like activity was observed for both bare CeO_2_ NPs and CeO_2_:Gd NPs. This type of enzyme-like activity was found to be pH-dependent. In a neutral medium (pH = 7.4), nanoscale CeO_2_ acted as a prooxidant enzyme (peroxidase), while in an alkaline medium (pH = 8.6), it lost its catalytic properties; thus, it cannot be regarded as a nanozyme. Both gadolinium doping and conjugation with a natural enzyme were shown to modulate the interaction of CeO_2_ NPs with the key components of redox homeostasis.

## 1. Introduction

Today, there is an urgent need for the development of theranostic agents combining both diagnostic and therapeutic properties, as well as advanced antioxidants capable of scavenging different types of free radicals [1,2,3]. This is especially true in the context of the treatment of complex diseases with heterogeneous expressions such as malignant tumors, where the use of standard clinical approaches for their diagnosis and treatment may be ineffective [4,5,6]. Metal oxide nanoparticles demonstrate impressive biological activity and are considered to be promising components for the creation of multifunctional theranostic agents [7,8,9]. These inorganic nanobiomaterials are characterized by a number of important physicochemical properties, such as ultrasmall particle size, high reactivity, biocompatibility and immunogenicity, which make them suitable for biomedical applications [4,10].

CeO_2_ nanoparticles (CeO_2_ NPs), which represent a new class of inorganic nanobiomaterial with enzyme-like activity (nanozymes), attract special attention. The increased interest of researchers in nanoscale CeO_2_ is due to its ability to self-regenerate and its multi-enzymatic activity [11,12,13]. CeO_2_ nanoparticles demonstrate superoxide dismutase- (SOD-) [14,15,16,17], catalase- [18,19,20], peroxidase- [21,22], oxidase- [23,24], phosphatase- [25], photolyase- [26], phospholipase- [27], nuclease- [28], haloperoxidase- [29], lipo-/phospholipoperoxidase- [30] and uricase-like [31] activities. The multi-enzymatic activity of nanoscale CeO_2_ is known to be strongly pH-dependent [23,32]. This enables switching between the anti- and prooxidant properties of CeO_2_ NPs, which is extremely important for biomedical applications.

The biological activity of CeO_2_ NPs can be affected by various factors [33,34], one of which is the surface oxidation state, which is believed to significantly affect the enzyme-mimetic activities of nanoscale CeO_2_ [35]. Numerous methods and approaches have been described for regulating the surface structure of inorganic nanomaterials [33,36]. Doping CeO_2_ NPs with the ions of transition and rare earth elements (REEs) is an approach of choice for obtaining multifunctional nanozymes based on nanoscale CeO_2_ for practical applications [33,37]. According to a widespread assumption, doping CeO_2_ NPs with REE ions (Eu, Er, Nd, Pr, La, Sm) leads to an increase in the concentration of oxygen vacancies and an increase in the Ce^3+^/Ce^4+^ ion ratio on the surface [38,39]. At the same time, there are controversial data in the literature on the effect of doping ceria with REE ions on its biological activity and catalytic characteristics while maintaining high oxygen non-stoichiometry [38,39,40,41].

Doping CeO_2_ NPs with Gd ions to obtain a new class of contrast agents for magnetic resonance imaging is of particular interest for the development of advanced hybrid theranostic agents [4,42]. Prospects for the creation of such hybrid nanobiomaterials based on nanoscale CeO_2_ are subject to the following considerations. On the one hand, the content of surface Ce^3+^ ions and the biocatalytic properties of CeO_2_ NPs can be further enhanced by modification with gadolinium ions [4,43]. On the other hand, there is an issue with using Gd-based complexes as contrast agents, due to the high toxicity associated with free gadolinium ions (those ions that are not incorporated into a crystal structure or part of a complex) [44,45]. In this regard, strategies to reduce the dosage of Gd are being actively developed to improve contrasting ability (relaxivity). To achieve this, nanoparticles containing Gd are produced [46,47] that are stabilized with biocompatible ligands [48,49] and conjugated with targeting biomolecules [50,51].

It should be noted that most studies have focused on the features of the surface state of CeO_2_ NPs when doped with Gd, and on some aspects of the biological activity of this type of material. At the same time, there remains a need for a comprehensive analysis of the multi-functional biomimetic activities of ceria-based nanozymes, especially those doped with gadolinium and other rare earth elements.

To date, some progress has been achieved in this direction. Composites based on gadolinium-doped CeO_2_ NPs (CeO_2_:Gd NPs) have been reported as being multifunctional agents for magnetic resonance imaging and computed tomography [38,39,52]. These composites have demonstrated improved sensitivity for tumor detection [38] and high *T*_1_ relaxivity [52], good biocompatibility [52] and stability in biological milieu [38]; they have also exhibited high enzyme-like activity [39] and selective cytotoxicity to cancer cells [52].

The current study is focused on a comparative analysis of the biomimetic activity and radical-scavenging properties (antioxidant activity) of CeO_2_:Gd NPs. The biochemical behavior of CeO_2_:Gd NPs was investigated in relation to the key components of redox homeostasis—superoxide anion radicals, hydrogen peroxide, and alkylperoxyl radicals. The biomimetic activities, namely SOD- and peroxidase-like activities, and radical-scavenging properties of CeO_2_:Gd NPs were assessed using a chemiluminescent method with selective probes: lucigenin, 8-amino-5-chloro-7-phenyl-pyrido [3,4-d]pyridazine-1,4(2*H*,3*H*)dione, and luminol, respectively. Both bare CeO_2_ NPs and CeO_2_:Gd NPs synthesized using an ion-exchange method followed by hydrothermal treatment demonstrated pronounced peroxidase-like biomimetic activity and radical-scavenging properties. Unexpectedly, bare CeO_2_ NPs and CeO_2_:Gd NPs showed no SOD-mimetic activity while not significantly inhibiting the activity of the natural superoxide dismutase enzyme in the NP-SOD conjugates. The results obtained may be of fundamental importance for understanding the features and mechanisms of the modulation of the nanozyme activity of CeO_2_ NPs by gadolinium doping and conjugation with SOD.

## 2. Materials and Methods

### 2.1. Materials (Chemicals)

The following reagents were used in this work: Ce(NO_3_)_3_·6H_2_O (LANHIT, Moscow, Russia, 99.9%), Gd(NO_3_)_3_·6H_2_O (Sigma Aldrich, St. Louis, MO, USA, 99.9%), NH_4_OH (Chimmed, Moscow, Russia, puriss. spec.), Amberlite IRA-410 anion exchange resin (Sigma Aldrich), NaOH (Sigma Aldrich, ≥98%), hydrochloric acid (SigmaTech, Arlington, VA, USA, puriss. spec.), ammonium citrate dibasic (Sigma Aldrich, ≥98%), isopropyl alcohol (Chimmed, puriss. spec.).

### 2.2. Methods for the Synthesis of Aqueous Sols of Cerium Dioxide, Including Those Doped with Gadolinium

The synthesis of aqueous cerium dioxide sols and ceria-gadolinia solid solutions was carried out in accordance with a previously described method [53]. First, the anion exchange resin (Cl form) was converted to the OH form. For this, the anion exchange resin was soaked in a 10% NaOH solution and stirred for one day, after which the supernatant liquid was removed and the resin was soaked in a new portion of the NaOH solution. This procedure was carried out for one week. Next, the resin was repeatedly washed with distilled water.

Anion exchange resin was gradually added to 100 mL of a 0.01 M solution of Ce(NO_3_)_3_∙6H_2_O (0.434 g), with stirring, to pH = 9.6. Then, the resin was separated by decantation. Next, the solution, in a Teflon autoclave, was incubated in a drying oven for 12 h at 190 °C.

The method for preparing aqueous sols of solid solutions of cerium dioxide doped with gadolinium was similar to the method for synthesizing aqueous sols of bare cerium dioxide. At the initial stage, a mixed solution of Ce(NO_3_)_3_·6H_2_O and Gd(NO_3_)_3_·6H_2_O was prepared at such concentrations that the molar content of gadolinium in the final product was 10% or 20%.

As a control in chemiluminescence measurements, a solution was used that was obtained by preparing a suspension of anion exchange resin (OH form) with pH 9.6, decanting the resin, and then subjecting the mother solution to hydrothermal treatment at 190 °C for 12 h.

### 2.3. Materials Characterisation

Powder X-ray diffraction analysis (XRD) of the samples was carried out on a Bruker D8 Advance diffractometer, Bruker, Billerica, MA, USA (CuKα radiation, θ–2θ geometry) in the angle range of 3–120° 2θ, with a step of 0.01–0.02° 2θ and a signal accumulation time of at least 0.3 s per point. JANA2006 software was used for full-profile analysis of diffraction patterns. The crystallite sizes were assessed using the Scherrer equation $D=\frac{180K\lambda }{\pi L_{x}}$ , where *D* is the crystallite size, *K* is the Scherrer constant, *λ* is the X-ray wavelength, and *L_x_* is the full-profile analysis parameter in JANA2006 software.

The determination of the atomic ratio of cerium and gadolinium in the solid state products obtained was carried out by Energy-dispersive X-ray spectroscopy (EDX) on an NVision 40 microscope (Carl Zeiss, Jena, Germany), using an X-MAX 80 mm^2^ energy-dispersive detector. The analysis was carried out at an accelerating voltage of 20 kV. The elemental composition was calculated in a semi-automatic mode, using INCA Oxford software (Oxford Instruments, Oxford, UK, version 2.1).

The microstructure of the samples was studied by transmission electron microscopy (TEM), using a Leo912 AB Omega electron microscope (Carl Zeiss, Jena, Germany) at an accelerating voltage of 100 kV. The samples were placed on copper grids with a diameter of 3.05 mm, coated with a polymer film. Transmission images were obtained at magnifications up to ×500,000.

UV-vis absorption spectra of CeO_2_ sols were recorded using a UV-vis-NIR spectrophotometer (Cary 5000, Agilent, Santa Clara, CA, USA) at room temperature. The absorption spectrum of distilled water was used as the baseline.

The particle size distribution was obtained using a Photocor Complex multi-angle dynamic light scattering spectrometer with a diode laser (λ = 650 nm, radiation power 25 mW). All measurements were carried out at a scattering angle of 90°. 

Raman spectra were recorded on a Renishaw InVia Reflex spectrometer (Renishaw, Dundee IL, USA) using a Renishaw 633 nm HeNe laser (radiation power 3 mV); spectrum accumulation time was 100 s and data were averaged over three spectra.

### 2.4. Preparation of Ceria-SOD Conjugates

A stock solution of Cu,Zn-superoxide dismutase (SOD, #S8160-15KU, Sigma, Kawasaki-shi, Japan) with an activity of 2400 U/mL (c = 25 µM) was prepared by quickly dissolving a sample of SOD in deionized water (18 MOhm cm). Conjugates of CeO_2_ NPs and CeO_2_:Gd NPs with SOD were prepared according to a previously described procedure [54]. Briefly, an aqueous solution of SOD with an activity of 100 U/mL (c = 1 μM) was mixed with 10 mM CeO_2_ sol or 10 mM CeO_2_:Gd sol. The mixtures were incubated for 60 min in the dark at room temperature.

### 2.5. Analysis of Enzyme-like Activity and Radical-Scavenging Properties

As a blank in chemiluminescent measurements, a reaction mixture without the analyzed sample was used. The blank includes a phosphate buffer solution, a radical initiator and a chemiluminescent probe.

#### 2.5.1. SOD-Mimetic Assay

To analyze the SOD-like activity of ceria sols, a chemiluminescence method was used, based on recording the chemiluminescence of lucigenin during its oxidation by superoxide anion radicals (•O_2_^−^). The generation of superoxide anion radicals occurs as a result of the oxidation of xanthine to uric acid in the presence of oxygen [55,56].

Aliquots of aqueous solutions of xanthine (20 μM, #X0626, Sigma), lucigenin (20 μM, #393824, J&K, San Jose, CA, USA), and the test sample were rapidly added to a cuvette with a phosphate buffer solution (100 mM, pH = 7.4). The background signal was recorded for 30–60 s, and then xanthine oxidase (*a* = 8.8 mU/mL, #X1875-25UN, Sigma) was added. The intensity of chemiluminescence was recorded at 37 °C on a 12-channel Lum-1200 chemiluminometer (DISoft, Moscow, Russia). All experiments were conducted in triplicate. The results were processed using PowerGraph software (version 3.3).

#### 2.5.2. Peroxidase Mimetic Assay

The peroxidase-like activity of ceria sols was analyzed with respect to hydrogen peroxide in the presence of L-012 (8-amino-5-chloro-7-phenyl-pyrido [3,4-d]pyridazine-1,4(2*H*,3*H*)dione), which is a highly sensitive luminol analogue [57,58].

Aliquots of H_2_O_2_ (20 μM, #H1009, Sigma) and L-012 (10 μM, #SML2236, Sigma) were rapidly added to a cuvette with a phosphate buffer solution (100 mM, pH = 7.4). The background signal was recorded for 60–90 s. An aliquot of the test sample was added after the chemiluminescence intensity reached a stationary value. All experiments were conducted in triplicate. Chemiluminescence was recorded on a 12-channel Lum-1200 chemiluminometer (DISoft, Russia). The results were processed using PowerGraph software (version 3.3).

The chemiluminescence curves were used for kinetic modeling and estimating the kinetic constants for the interaction of ceria nanoparticles with the substrate. Mathematical modeling of chemiluminograms was carried out using the Kinetic Analyzer software (http://www.powergraph.ru/soft/) [55,59].

#### 2.5.3. Analysis of Radical-Scavenging Properties

To analyze the radical scavenging properties of ceria sols, a model reaction of the generation of alkylperoxyl radicals (ROO•) during the decomposition of 2,2′-azobis(2-amidinopropane) dihydrochloride (AAPH) was used [60].

AAPH (2.5 μM, #123072, Sigma) and luminol (2.0 μM, #123072, Sigma) were rapidly added to a cuvette thermostated at 37 °C with a phosphate buffer solution (100 mM, pH = 7.4). An aliquot of the test sample was added after the chemiluminescence intensity reached a stationary value. Chemiluminescence was recorded on a 12-channel Lum-1200 chemiluminometer (DISoft, Russia).

#### 2.5.4. Statistical Analysis

All experiments were conducted in triplicate. The experimental results of enzyme-like and radical-scavenging activities were processed using PowerGraph software (version 3.3). Cytotoxicity analysis and cell culture experiments were processed using GraphPad Prism 8 software (version 8.0). The MTT assay data are presented as M ± SD. The statistical significance of the deviations between the test sets and the control was confirmed using the Mann-Whitney U test. Images were processed using Adobe Photoshop CC software 2017.

## 3. Results and Discussion

### 3.1. Characterisation of CeO_2_ NPs and Gadolinium-Doped CeO_2_ NPs

**Figure 1:**
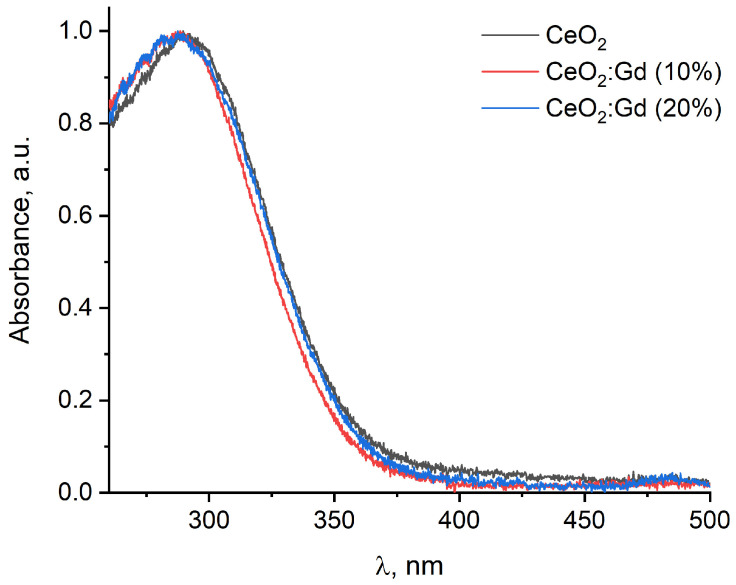
Electron absorption spectra of cerium dioxide sols with different levels of gadolinium doping.

Three transparent solutions were obtained through anion exchange treatment of aqueous solutions of cerium and gadolinium (0%, 10%, 20%) nitrates followed by hydrothermal synthesis, in which a distinct Tyndall cone was observed. The concentration of sols was 0.01 M, as determined by the thermogravimetric method. Figure 1 shows the UV-vis absorption spectra of CeO_2_ sols with different levels of Gd doping.

The UV-vis spectra of both the bare CeO_2_ NPs and CeO_2_:Gd NPs are typical of nanocrystalline cerium dioxide and contain a broad absorption band with a maximum in the region of 290–300 nm (Figure 1). The determined band gap values for all samples were nearly 3.4 and 3.8 eV for indirect and direct transitions, respectively, which agree with previously reported data [53,61].

According to dynamic light scattering data, the hydrodynamic radii of the particles in ceria sols were generally close to one another, measuring about 10–15 nm.

**Figure 2:**
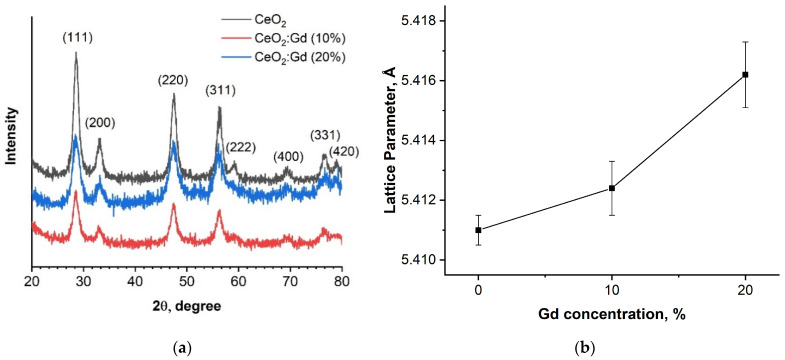
(**a**) Diffractograms of CeO_2_ samples with different levels of Gd doping; (**b**) dependence of the unit cell parameters on the nominal gadolinium content.

Ceria sols dried at 60 °C were analyzed by XRD. As revealed by the XRD patterns, the samples of bare CeO_2_ NPs and CeO_2_:Gd NPs (Figure 2a) were single-phase cerium dioxide with a fluorite structure (PDF2 No. 34-394), which is in good agreement with the literature [33,62,63]. At gadolinium concentrations of up to 20%, no gadolinium-containing impurity phases were detected in the obtained samples, which indirectly confirms the inclusion of Gd cations in the CeO_2_ structure.

Diffraction maxima at 28.5°, 33.0°, 47.5°, 56.4°, 59.1°, 69.4°, 76.6°, and 79.1° correspond to (111), (200), (220), (311), (222), (400), (331), and (420) CeO_2_ crystallographic planes, respectively (Figure 2a) [33,64]. The unit cell parameters calculated using a full profile analysis of diffraction patterns are presented in Figure 2b. The unit cell parameter increased almost linearly, which was caused by the incorporation of a Gd^3+^ ion in the CeO_2_ structure, since the ionic radius of Gd^3+^ (0.107 nm) is larger than the ionic radius of Ce^4+^ (0.097 nm) [29,33,37,62,65].

According to EDX data, the average Ce:Gd ratio in gadolinium-doped ceria samples was 89.6:10.4 and 77.9:22.1, which is very close to the nominal gadolinium content (10% and 20%). This further confirms the successful synthesis of solid solutions of CeO_2_:Gd (10%, 20%) by the ion-exchange method followed by hydrothermal treatment.

**Figure 3:**
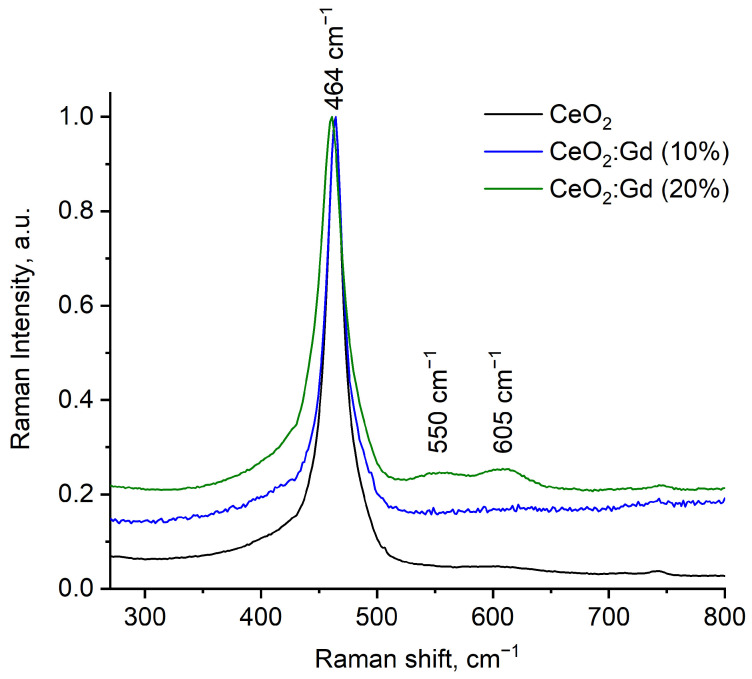
Raman spectra of dried aqueous cerium dioxide sols with different levels of gadolinium doping.

Figure 3 shows the Raman spectra of bare CeO_2_ NPs and CeO_2_:Gd NPs.

The spectra of all the samples show a characteristic Raman peak at 464 cm^−1^, corresponding to symmetric vibrations of oxygen ions in CeO_8_ octahedra [33,66,67]. For CeO_2_:Gd NPs, a shift of the Raman peaks to lower energies and their broadening can be observed, in comparison with bare CeO_2_ NPs, which can be explained by an increase in the unit cell parameter, including that caused by doping [66,68,69]. At a 20% doping level, peaks at 550 cm^−1^ and 605 cm^−1^ appear in the Raman spectrum. This reflects an increase in the concentration of oxygen vacancies and provides further evidence for the formation of solid solutions [66,70].

**Figure 4:**
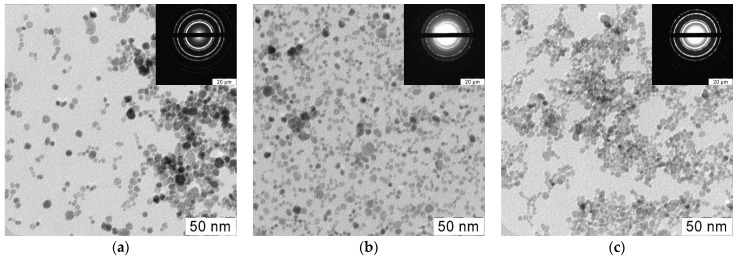
TEM images of CeO_2_ nanoparticles with different levels of gadolinium doping (**a**) 0%, (**b**) 10%, (**c**) 20% (Insets: electron diffraction data).

**Figure 5:**
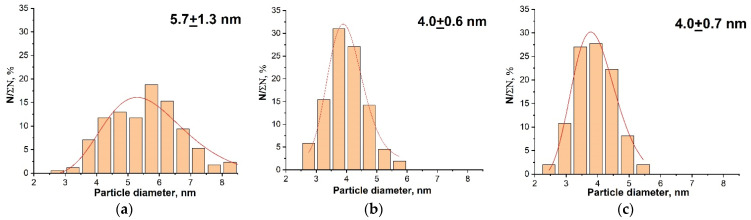
Particle size distributions in CeO_2_ sols with different levels of gadolinium doping: (**a**) 0%, (**b**) 10%, (**c**) 20%.

TEM and electron diffraction data (Figure 4) indicate that the introduction of gadolinium had little effect on the shape and size of CeO_2_ particles (Figure 5). CeO_2_ and CeO_2_:Gd nanoparticles have almost the same size, which is ~5 nm; there were no signs of segregation of the dopant on the surface of the crystallites, which could limit their growth. Both individual CeO_2_:Gd NPs and their aggregates are characterized by a fairly small size. This makes the biomedical application of such particles possible, similar to bare CeO_2_ NPs.

TEM and electron diffraction data (strong ring patterns corresponding to (111) and (220) crystal planes) confirm the results of powder X-ray diffraction analysis (Figure 2a). SAED patterns did not show the presence of any impurity gadolinium phases, which is consistent with the literature data concerning gadolinium-doped ceria formation under hydrothermal conditions [33].

### 3.2. Enzyme-like Activity and Radical-Scavenging Properties of CeO_2_ NPs and Gadolinium-Doped CeO_2_ NPs

A heterovalent doping is a convenient tool for changing redox-related properties and oxygen mobility in inorganic nanomaterials. Doping nanoscale CeO_2_ with trivalent lanthanides leads to modification of its biocatalytic activity and opens up wide possibilities for its tuning. In a number of recent studies, doping cerium dioxide with REE ions (Eu, Nd, Pr, La, Sm, Er) was shown to lead to an increase in the concentration of oxygen vacancies, a change in the Ce^3+^/Ce^4+^ ion ratio, and an increase in the catalytic activity and bioactivity of cerium dioxide [40,43]. At the same time, in some reports, a decrease in the biochemical activity of CeO_2_ nanoparticles was observed when they were doped with REE ions while maintaining their high oxygen non-stoichiometry [29,71,72].

In the present study, efforts were focused on evaluating the effect of gadolinium doping on the biomimetic activity, namely SOD- and peroxidase-like activities, and the radical-scavenging properties (antioxidant activity) of nanoscale CeO_2_.

#### 3.2.1. SOD-Mimetic Activity

The SOD-like activity of nanomaterials was analyzed using the chemiluminescent method. Measurements were carried out in the presence of lucigenin, a probe sensitive to the presence of superoxide anion radicals (•O_2_^−^) [73,74,75].

In the present study, an analysis was made of both the biochemical behavior of bare CeO_2_ NPs and CeO_2_:Gd NPs in relation to •O_2_^−^ and the activity of the bare CeO_2_ NPs and CeO_2_:Gd NPs with SOD conjugates.

**Figure 6:**
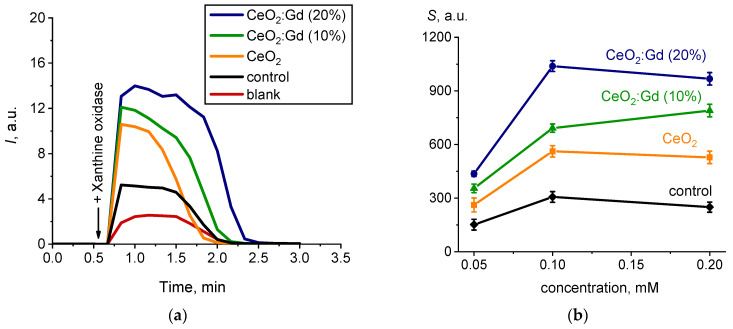
(**a**) Chemiluminograms characterizing the SOD-like activity of bare CeO_2_ NPs, CeO_2_:Gd NPs (10% and 20%) and a control sample; sample concentration 0.2 mM; (**b**) light sums (*S*) as a function of the concentration of samples.

Figure 6a shows chemiluminograms recorded upon adding xanthine oxidase to reaction mixtures containing xanthine, lucigenin and CeO_2_ sols (including Gd-doped CeO_2_ sols).

The addition of xanthine oxidase to a reaction mixture containing a substrate, a chemiluminescent probe, and doped or undoped ceria sols led to the appearance of luminescence due to the formation of superoxide anion radicals. Interestingly, bare CeO_2_ NPs synthesized using the ion-exchange method with subsequent hydrothermal treatment did not exhibit SOD-like activity, unlike nanoscale CeO_2_ synthesized by other methods [14,15,54,55]. The enhancement of lucigenin-activated chemiluminescence, compared with the control level of luminescence (blank) in colloidal solutions containing CeO_2_:Gd NPs, also indicated the absence of SOD-like activity (Figure 6a). Thus, both bare CeO_2_ NPs and CeO_2_:Gd NPs were pro-oxidants and contributed to the generation of reactive oxygen species (ROS).

**Figure 7:**
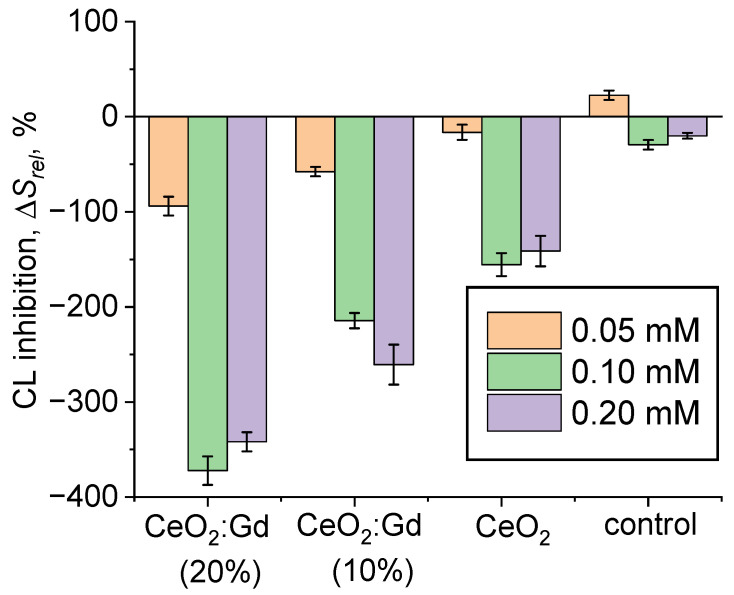
Histograms of the relative degrees of chemiluminescence suppression (Δ*S_rel_*_._) for bare CeO_2_ NPs, CeO_2_:Gd NPs (10% and 20%) and a control sample. Δ*S_rel_*_._ values were normalized to 100%.

Figure 6b shows the experimental dependences of the integral parameter *S* (the area under the chemiluminescence curve, which is proportional to the number of free radicals generated) for various concentrations of ceria sols. The relative degree of chemiluminescence suppression Δ*S_rel._* was calculated to compare the prooxidant effects inherent in ceria sols (Figure 7). The following formula was used for the calculation of $\Delta S_{rel.}$ values:

$$\Delta S_{rel.},\;\%\;=\frac{(S_{0}-S)\;}{S_{0}}\times 100\%,$$

where *S*_0_ and *S* are light sums (the area under the chemiluminescence curve) for the control experiment (without the addition of ceria sols) and for the experiment with ceria sols added.

The generation of ROS was enhanced in the presence of ceria sols. This effect was most pronounced in the case of CeO_2_:Gd NPs (Figure 7). An increase in dopant concentration caused an increase in the pro-oxidant properties of nanoscale CeO_2_: bare CeO_2_ < CeO_2_:Gd (10%) < CeO_2_:Gd (20%).

**Figure 8:**
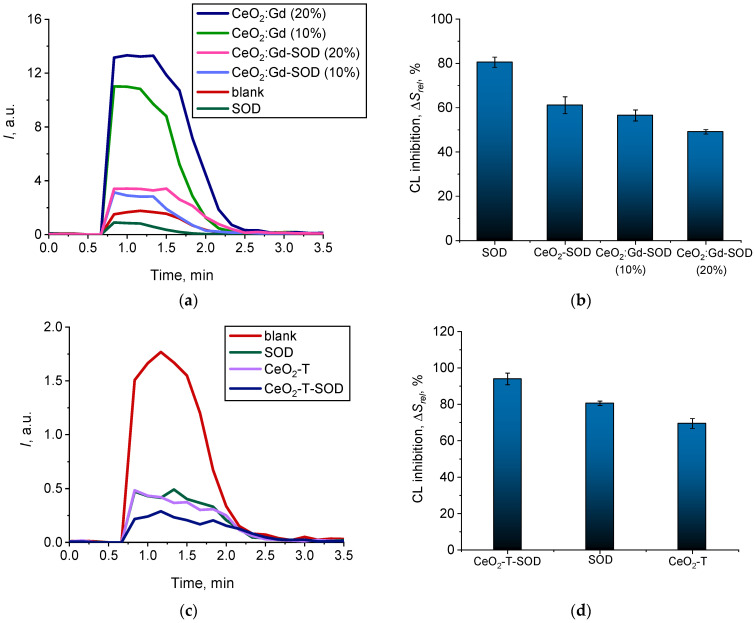
(**a**) Chemiluminograms characterizing the SOD-like activity of CeO_2_:Gd NPs (10% and 20%, 0.10 mM), ceria-SOD conjugates (CeO_2_-SOD, CeO_2_:Gd-SOD (10%), CeO_2_:Gd-SOD (20%)) and SOD (10 nM); (**b**) histograms of the relative degrees of chemiluminescence suppression (Δ*S_rel._*) for CeO_2_:Gd NPs, ceria-SOD conjugates and SOD; (**c**) chemiluminograms characterizing the SOD-like activity of citrate-stabilized CeO_2_ NPs (CeO_2_-T, 0.10 mM), ceria-SOD conjugate (CeO_2_-T-SOD) and SOD (10 nM); (**d**) histograms of the relative degrees of chemiluminescence suppression (Δ*S_rel._*) for citrate-stabilized CeO_2_ NPs, ceria-SOD conjugate and SOD. Δ*S_rel._* values were normalized to 100%.

Figure 8a shows chemiluminograms that were registered upon adding xanthine oxidase to reaction mixtures containing xanthine, lucigenin, and conjugates of CeO_2_:Gd NPs with SOD. According to these data, both bare CeO_2_ NPs and conjugates of CeO_2_:Gd NPs with SOD possessed SOD-like activity (Figure 8a,b). Thus, SOD can be conjugated to gadolinium-doped CeO_2_ NPs without the loss of its own enzymatic activity. Moreover, the SOD-like activity of both bare CeO_2_ NPs and conjugates of CeO_2_:Gd NPs with SOD was less than the activity of pristine SOD (Figure 8b). Increasing the Gd concentration in nanoparticles led to a decrease in the SOD-like activity of conjugates of CeO_2_:Gd NPs with SOD: CeO_2_-SOD > CeO_2_:Gd-SOD (10%) > CeO_2_:Gd-SOD (20%) (Figure 8b). The observed trend in changes in SOD-like properties corresponds to a concept reported in a number of earlier papers, according to which excessive dopant concentration can cause a decrease in the biocatalytic activity of nanoscale CeO_2_ [76].

Since bare CeO_2_ NPs obtained by the anion exchange method, contrary to expectations, did not demonstrate SOD-like activity, a citrate stabilized CeO_2_ sol (the molar ratio of ceria and citrate was 1:1; particle size ~3 nm) synthesized by thermal hydrolysis of ammonium cerium(IV) nitrate was chosen as a reference sample for the preparation of conjugates with SOD [77]. Figure 8d shows chemiluminograms that were recorded for the citrate-stabilized CeO_2_ sol and its conjugate with SOD.

The data obtained confirm the SOD-like activity of the citrate-stabilized CeO_2_ sol (Figure 8c). The conjugate of citrate-stabilized CeO_2_ NPs with SOD was characterized by more pronounced enzyme-like activity, effectively inhibiting superoxide anion radicals, compared with non-functionalized ceria (Figure 8d). This indicates a synergistic effect of citrate-stabilized CeO_2_ NPs and SOD. These results correlate well with data found in the literature [54,55,78]. A number of previous studies have shown that the SOD-like activity of CeO_2_ NPs increases significantly after their interaction with Cu,Zn-SOD or the electron donor molecule [Ru(dcbpy)_2_(NCS)_2_] [54,78]. Thus, the conjugation of CeO_2_ NPs with the antioxidative enzyme is not only a means of increasing the SOD-like activity of nanoscale CeO_2_ but also an effective approach to obtaining nanomaterials with tunable enzyme-like activity.

Along with functionalizing the surface of nanoscale CeO_2_ with biologically active molecules, an important method for improving its biocatalytic activity involves doping CeO_2_ NPs with ions of allovalent transition and rare earth elements [33,52,62,79]. This leads to the formation of oxygen vacancies for local charge compensation. A common assumption is that, under physiological conditions, ROS can be catalytically degraded through the Ce^3+^/Ce^4+^ redox pair [62]. The chemical doping of nanoceria can significantly improve the catalytic activity of the Ce^3+^/Ce^4+^ redox pair toward biochemically relevant ROS [29,37,80]. In this regard, a high proportion of surface Ce^3+^ ions is important in the use of nanobiomaterials based on CeO_2_ in the treatment of diseases caused by disturbances of redox homeostasis [80,81,82].

A superoxide anion radical (•O_2_^−^) is one of the primary ROS formed during free radical metabolism in living organisms. The main sources of •O_2_^−^ in the body are mitochondria and enzymatic systems: NADPH oxidase, xanthine oxidase, lipoxygenase, and cyclooxygenase [74,83,84,85]. The ability to catalyze the dismutation of superoxide anion radicals was among the first types of enzyme-like activity discovered in nanocrystalline CeO_2_ [14,15,86,87]. Doping CeO_2_ NPs with REE ions (La^3+^, Sm^3+^, Er^3+^) was found to result in a significant increase in the SOD-like activity of nanoscale CeO_2_, which was associated with an increase in the concentration of Ce^3+^ ions [43]. Presumably, the rate of inhibition of superoxide anion radicals of CeO_2_ NPs doped with REE ions is closely related to the concentration of Ce^3+^ ions [33,88,89].

In a recent study, a promising endothelial protection strategy for the effective therapy of atherosclerosis through the use of CeO_2_:Gd NPs was demonstrated [62]. The resulting CeO_2_:Gd nanozymes combined inactivation capabilities for a broad spectrum by ROS and significantly reduced intracellular oxidative stress caused by oxidized low-density lipoprotein [62]. The effect of dopant concentration on the SOD- and catalase-like activity of nanoscale CeO_2_ was studied. The improvement in the enzyme-like properties of nanoscale CeO_2_ with an increase in the level of Gd doping was explained by the formation of defects in the form of oxygen vacancies and an increase in the proportion of surface Ce^3+^ ions [29,65,81]. In another study, a two-stage mechanism was proposed to explain the enhancement of the SOD-like activity of nanoscale CeO_2_ with Gd doping [33]. At the first stage, the enzymatic dismutation of superoxide anion radicals occurs, with the formation of hydrogen peroxide. At the second stage, the regeneration of Ce^3+^ ions occurs, with electronic transfer and the formation of oxygen vacancies [33].

Thus, according to a widespread assumption, the biochemical activity of CeO_2_ NPs, including those doped with rare earth ions, is caused exclusively by redox transitions between the Ce^3+^/Ce^4+^ states [14,15,90]. It should be noted, however, that the actual oxidation state of cerium in nanocrystalline CeO_2_ is currently the subject of extensive debate, since there are reasonable grounds for questioning the presence of trivalent cerium in nanoscale CeO_2_ [91,92].

#### 3.2.2. Peroxidase Mimetic Activity

The redox activity of bare CeO_2_ NPs and CeO_2_:Gd NPs was analyzed in relation to one of the key molecules of free radical homeostasis, namely hydrogen peroxide. To assess the peroxidase-like activity of the samples using the chemiluminescent method, a highly sensitive analogue of luminol—8-amino-5-chloro-7-phenyl-pyrido [3,4-d]pyridazine-1,4(2*H*,3*H*)dione (L-012)—was chosen as a chemiluminescent probe molecule [57,58].

**Figure 9:**
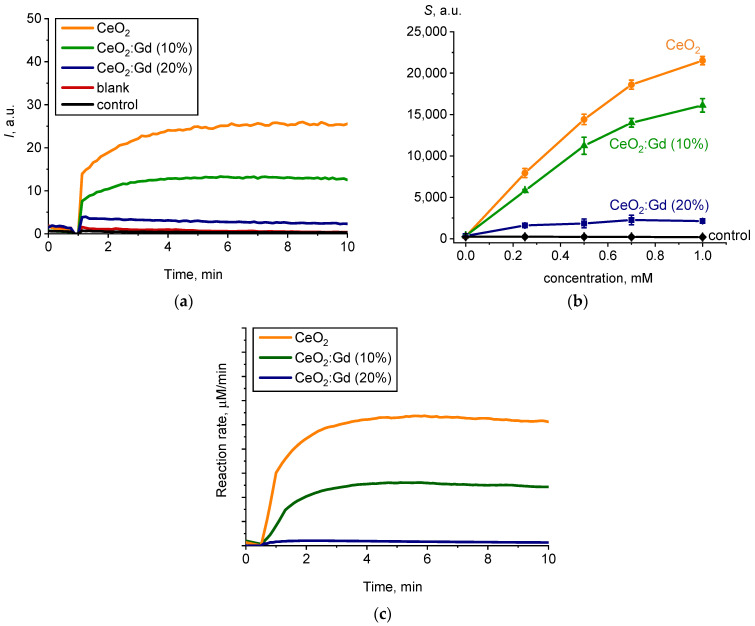
(**a**) Chemiluminograms characterizing peroxidase-like activity of the bare CeO_2_ NPs, CeO_2_:Gd NPs (10% and 20%) and a control sample at pH 7.4, sample concentration 0.5 mM; (**b**) light sums (*S*) as a function of sample concentrations; (**c**) the results of the mathematical modeling of the corresponding chemiluminograms.

Figure 9a shows chemiluminograms that were recorded when bare CeO_2_ NPs and CeO_2_:Gd NPs were added to a reaction mixture containing L-012 and H_2_O_2_ (pH = 7.4). In the presence of CeO_2_-based nanozymes, the luminescence intensity of the L-012 oxidation product increased.

The appearance of chemiluminescence kinetic curves with a luminescence intensity reaching a stationary level enables the conclusion that the samples demonstrated peroxidase-like properties (Figure 9a). The experimental dependences of the integral index *S* (the area under the chemiluminescence curve, which is proportional to the level of free radicals generated) on the concentration of the samples (Figure 9b) demonstrate the dose-dependent nature of the enhancement of chemiluminescence in the presence of CeO_2_-based nanozymes. An increase in the level of Gd doping caused a decrease in the peroxidase-like activity of CeO_2_ at pH = 7.4 (Figure 9b): bare CeO_2_ > CeO_2_:Gd (10%) > CeO_2_:Gd (20%).

To confirm the enzyme mimetic property of CeO_2_ NPs and to estimate the catalytic constants, the mathematical modeling of chemiluminograms was performed (Figure 9c) [55,59].

As a possible mechanism for the peroxidase-like activity of ceria nanoparticles, the following model was used:

H_2_O_2_ + CeO_2_ → C_1_ (1)

C_1_ + L-012 → C_2_ + L-012• (2)

C_2_ + L-012 → CeO_2_ + L-012• (3)

L-012• + H_2_O_2_ → P + hν (4)

where C_1_ and C_2_—reaction intermediates 1 and 2, L-012•—the oxidation product of L-012, P—chemiluminescence reaction product.

Kinetic modeling showed that the above model agrees well with the experimental data (Figure 9a,c). The estimated rate constants of reactions (1)–(4) are shown in Table 1.

The constant *k*_4_ was chosen as the key kinetic parameter for comparing the peroxidase-like activity of the samples, as the chemiluminescent technique is based on the registration of the luminescence of the reaction product (Equation (4)). The data presented in Table 1 show that doping bare CeO_2_ NPs with 10% and 20% Gd leads to a decrease in the catalytic activity of CeO_2_ NPs toward hydrogen peroxide by 2.5 and 3.7 times, respectively.

This is consistent with the previously described assessment of the catalytic activity of CeO_2_:Gd NPs toward the oxidation of hydrogen peroxide to hydroxyl radicals [29]. Doping with Gd was found to reduce the activity of catalytic sites on the surface of CeO_2_ NPs. In a neutral medium (pH = 7.4), CeO_2_:Gd NPs catalyzed the decomposition of H_2_O_2_ and the formation of •OH through a Fenton-like reaction mechanism [29]. Based on these results, the authors suggested that increasing the proportion of surface Ce^3+^ ions in CeO_2_:Gd NPs could effectively increase the density and content of active sites in the NP catalyst [29]. It is important to note that Gd^3+^ dopants are not directly involved in the oxidation of H_2_O_2_, due to the high stability of the Gd^3+^ oxidation state [29]. The tendency for catalytic activity to decrease with increasing dopant concentration in CeO_2_:Gd NPs was also observed in a study of the scavenging activity of nanoscale CeO_2_ against hydroxyl radicals [72]. It was suggested that the redox inactivity of gadolinium(III) in the crystal lattice of nanoscale CeO_2_ slows the redox cycling of Ce^3+^/Ce^4+^, causing a deterioration in the antioxidant properties of CeO_2_:Gd NPs [72]. These data are confirmed by other reports, in which researchers explain such a dependence as being the result of the formation of stable oxygen vacancy defect complexes [41].

**Figure 10:**
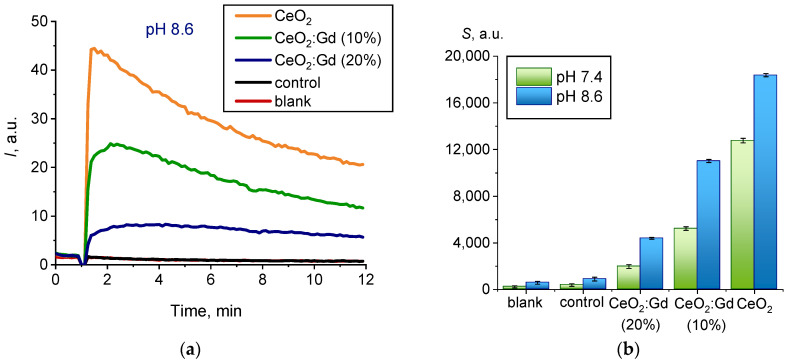
(**a**) Chemiluminograms characterizing peroxidase-like activity of the bare CeO_2_ NPs and CeO_2_:Gd NPs (10% and 20%) at pH = 8.6, sample concentration 0.5 mM; (**b**) corresponding dependencies of light sums (*S*) at pH = 7.4 and 8.6.

Figure 10a shows chemiluminograms recorded when bare CeO_2_ NPs and CeO_2_:Gd NPs were added to a reaction mixture containing L-012 and H_2_O_2_ (pH = 8.6).

The appearance of the chemiluminescence kinetic curves suggests that, at pH = 8.6, bare CeO_2_ NPs and CeO_2_:Gd NPs (Figure 10a) demonstrate non-peroxidase-like activity toward hydrogen peroxide, when compared with the results obtained at pH = 7.4 (Figure 9a). Trends in changes in the biochemical activity of CeO_2_ nanoparticles caused by doping with gadolinium remained unchanged at all pH values studied (Figure 10b): bare CeO_2_ > CeO_2_:Gd (10%) > CeO_2_:Gd (20%).

It is known that nanoscale CeO_2_ in biological systems can exhibit both pro- and antioxidant activity toward hydrogen peroxide [59,93]. This is also typical for CeO_2_:Gd NPs. In a number of studies, it was found that CeO_2_:Gd NPs exhibit predominantly catalase-like activity toward hydrogen peroxide [39,62]. With increasing dopant concentration, the catalase-like properties of CeO_2_:Gd NPs have been found to increase [62]. The pH is one factor that significantly affects the balance of the pro- and antioxidant properties of CeO_2_ NPs. A similar pH dependence of biochemical activity has been found for CeO_2_ nanoparticles doped with Gd [39]. For example, for PEGylated Yb_2_O_3_:Gd@SiO_2_@CeO_2_, it has been shown that pH = 4.0 is the most optimal pH for the manifestation of catalase-like properties [39]. This is consistent with the data published earlier for bare CeO_2_ NPs [19,39].

It is particularly important to emphasize that, currently, the enzyme-like nature of the catalytic activity of inorganic nanomaterials is being questioned. A growing number of publications are highlighting the need to reconsider the correctness of using the formalism of enzymatic reactions when describing the activity of nanomaterials with respect to various model substrates [94,95]. All of the abovementioned issues definitely require systematic research based on methods and approaches used in enzymology and the kinetic analysis of reactions.

#### 3.2.3. Radical-Scavenging Properties (Antioxidant Activity)

**Figure 11:**
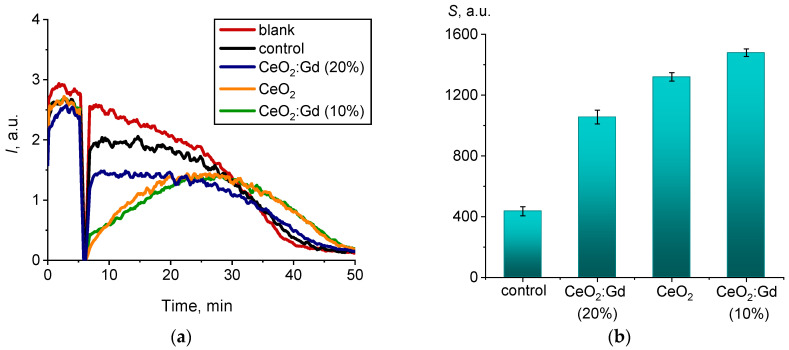
(**a**) Chemiluminograms characterizing the radical-scavenging properties of bare CeO_2_ NPs, CeO_2_:Gd NPs (10% and 20%) and a control sample, (sample concentrations 1.5 mM); (**b**) histograms of chemiluminescence suppression (*S_CL_*, a.u.) for ceria sols.

The radical-scavenging properties (antioxidant activity) of bare CeO_2_ NPs and CeO_2_:Gd NPs were analyzed in relation to organic radicals, namely alkylperoxyl radicals. The corresponding chemiluminograms of ceria sols are presented in Figure 11a. Note that, in Figure 11a, ceria samples possess different reaction kinetics. When bare CeO_2_ NPs and CeO_2_:Gd NPs (10%) were added, the luminescence level decreased to zero and then gradually increased (green and orange lines in Figure 11). Upon the addition of CeO_2_:Gd NPs (20%), the luminescence intensity instantly recovered to a reasonable level, which was lower than that of a blank. The differences in the rate of the restoration of luminescence intensity reflect the different radical-scavenging properties of the nanoparticles.

When bare CeO_2_ NPs and CeO_2_:Gd NPs interact with alkylperoxyl radicals in the presence of luminol, a decrease in luminescence intensity is observed. The different time dependences of chemiluminescence intensity indicate different values of the rate constants relating to the interaction of CeO_2_-based nanozymes with radicals [96]. The lowest degree of luminescence suppression was associated with the CeO_2_ sol doped with 20% Gd (Figure 11a). This effect is characteristic of weak antioxidants whose rate constant of radical scavenging is less than the rate constant of the interaction of radicals with luminol [96]. In turn, CeO_2_ sol with less Gd doping (10%) showed more pronounced antioxidant activity, which is characteristic of medium-strength antioxidants. The use of mathematical modeling in the study showed that the values of the rate constants for the interaction of medium-strength antioxidants with radicals were close to the value of the rate constant for luminol [96].

The area of the luminescence suppression region (*S_CL_*, a.u.) was calculated from chemiluminograms to quantitatively characterize the radical-scavenging properties of the sols, reflecting antioxidant capacity as defined by the degree of luminescence suppression, which is proportional to the number of scavenged radicals (Figure 11b). Both bare CeO_2_ sol and CeO_2_ sol doped with Gd (10%) demonstrated the most pronounced antioxidant properties against alkylperoxyl radicals. An increase in the dopant content to 20% led to a decrease in the radical-scavenging properties of nanodispersed cerium dioxide. A similar effect, whereby an excessive dopant concentration can cause a decrease in the biocatalytic activity of CeO_2_ nanoparticles, has been reported in some previous works [76].

### 3.3. Analysis of the Biological Activity of CeO_2_ and Gadolinium Doped (20%) CeO_2_ Sols, Including Sols Stabilized with Maltodextrin and Citrate Ions

One of the key factors for assessing the prospects for biomedical applications of engineered nanomaterials is their biological activity and the absence of toxicity. In this regard, the research team conducted an analysis of the biological activity and an assessment of the toxicity of aqueous sols of cerium dioxide, including sols doped with gadolinium (with a maximum doping level of 20%). Similar sols containing ammonium citrate (Cit) and maltodextrin (M)—stabilizers widely used in biomedical applications—were also analyzed. The concentration of stabilizers was taken in a twofold molar excess relative to the concentration of cerium oxide. The materials and methods for these measurements are presented in detail in Supplementary Materials.

According to the data obtained, CeO_2_ NPs or CeO_2_:Gd NPs (20%), including those stabilized with maltodextrin or ammonium citrate, had a high degree of biocompatibility in a culture of mouse fibroblast cells of the NCTC L929 line, as confirmed by a high level of mitochondrial membrane potential, high metabolic activity, and a low percentage of non-viable cells after 24 h of incubation, in a wide range of ceria-based sol concentrations (0.001–0.5 mg/mL) (Figures S1–S5, Supplementary Materials).

The high degree of biocompatibility of the synthesized ceria sols correlates well with previously obtained data on the high degree of biocompatibility of cerium-containing nanomaterials both in vitro [97] and in vivo [98].

## 4. Conclusions

In this study, stable colloids of bare CeO_2_ NPs and CeO_2_:Gd NPs containing various concentrations of gadolinium (10 mol.% or 20 mol.%) were synthesized. Unexpectedly, neither the bare CeO_2_ NPs nor the CeO_2_:Gd NPs demonstrated SOD-like activity, while they acted as prooxidants and contributed to the generation of ROSs. Gadolinium doping increased the prooxidant properties of nanocrystalline ceria. At the same time, conjugates of bare CeO_2_ NPs and CeO_2_:Gd NPs with SOD exhibited SOD-like activity. The SOD-like activity of the conjugates increased with a decrease in the gadolinium content in nanoscale CeO_2_.

In contrast to SOD-like properties, peroxidase-like activity was inherent in both bare CeO_2_ NPs and CeO_2_:Gd NPs. This type of enzyme-like activity was found to be pH-dependent. In neutral media (pH 7.4), nanoscale CeO_2_ acted as a prooxidant enzyme (peroxidase), while in slightly alkaline media (pH = 8.6), it lost its catalytic properties and thus cannot be regarded as a nanozyme. The peroxidase-like activity of nanoscale CeO_2_ increased with a decrease in gadolinium content.

Both CeO_2_ NPs and CeO_2_:Gd NPs demonstrated radical-scavenging properties toward organic (alkylperoxyl) radicals. The level of doping of nanoscale CeO_2_ with gadolinium affected the kinetics of the interaction of CeO_2_ NPs with alkylperoxyl radicals. Nanoscale CeO_2_ with a 10% Gd doping level acted as an antioxidant of medium strength, while nanoscale CeO_2_ with a 20% Gd doping level acted as a weak antioxidant (antioxidant of prolonged action).

Thus, doping nanoscale CeO_2_ with gadolinium and conjugation with SOD can be convenient tools for fine-tuning the biochemical behavior of CeO_2_ NPs. The data obtained can contribute to understanding the mechanisms responsible for the multifaceted nanozyme activity of CeO_2_ NPs and expand the possibilities for their biomedical application.

## Figures and Tables

**Table 1:** Rate constants of H_2_O_2_-L-012 oxidation reactions in the presence of cerium dioxide at pH = 7.4.

Sample	*k*_1_ (μM/min)	*k*_2_ (μM/min)	*k*_3_ (μM/min)	*k*_4_ (μM/min)
bare CeO_2_ NPs	3.0 × 10^−14^	3.5 × 10^9^	9.0 × 10^11^	9.8 × 10^−8^
CeO_2_:Gd NPs (10%)	9.2 × 10^−14^	8.7 × 10^9^	3.8 × 10^11^	3.9 × 10^−8^
CeO_2_:Gd NPs (20%)	9.0 × 10^−14^	9.5 × 10^9^	4.0 × 10^11^	2.6 × 10^−8^

## Data Availability

Data are contained within the article and Supplementary Materials.

## Supplementary Materials

The following supporting information can be downloaded at: https://www.mdpi.com/article/10.3390/nano14090769/s1, Figure S1. Assessment of the dehydrogenase activity level of NCTC L929 cells after 24 h of cultivation with bare CeO_2_ NPs or CeO_2_:Gd NPs (20%), including those stabilised with maltodextrin or ammonium citrate. Data are presented as M ± SD. The reliability of the results was calculated using the Mann-Whitney U test at *p* < 0.05. Figure S2. Microimages of the NCTC cell line L929 after staining with a mixture of dyes SYTO 9 (green)/propidium iodide (red) after 24 h of cultivation with bare CeO_2_ NPs, including those containing maltodextrin or ammonium citrate. The concentration is expressed in mg/mL. Scale bar is 100 microns. Figure S3. Microimages of the NCTC cell line L929 after staining with a mixture of dyes SYTO 9 (green)/propidium iodide (red) after 24 h of cultivation with CeO_2_:Gd NPs (20%), including those stabilised with maltodextrin or ammonium citrate. The concentration is expressed in mg/mL. Scale bar is 100 microns. Figure S4. Microimages of the NCTC cell line L929 after staining with tetramethylrhodamine (TMRE) after 24 h of cultivation with bare CeO_2_ NPs, including those stabilised with maltodextrin or ammonium citrate. Scale bar is 100 microns. Figure S5. Microimages of the NCTC cell line L929 after staining with tetramethylrhodamine (TMRE) after 24 h of cultivation with CeO_2_:Gd NPs (20%), including those stabilised with maltodextrin or ammonium citrate. Scale bar is 100 microns. Ref. [99] is cited in the Supplementary Materials.
