# Correction: Sozarukova et al. Gadolinium Doping Modulates the Enzyme-like Activity and Radical-Scavenging Properties of CeO_2_ Nanoparticles. *Nanomaterials* 2024, *14,* 769

**DOI:** 10.3390/nano15181429

**Published:** 2025-09-17

**Authors:** Madina M. Sozarukova, Taisiya O. Kozlova, Tatiana S. Beshkareva, Anton L. Popov, Danil D. Kolmanovich, Darya A. Vinnik, Olga S. Ivanova, Alexey V. Lukashin, Alexander E. Baranchikov, Vladimir K. Ivanov

**Affiliations:** 1 Kurnakov Institute of General and Inorganic Chemistry of the Russian Academy of Sciences, 119991 Moscow, Russia; 2 Materials Science Department, Lomonosov Moscow State University, 119234 Moscow, Russia; 3 Institute of Theoretical and Experimental Biophysics of the Russian Academy of Sciences, 142290 Pushchino, Russia; 4 Frumkin Institute of Physical Chemistry and Electrochemistry of the Russian Academy of Sciences, 119071 Moscow, Russia

In the original publication [1], there was an error in Figures S2–S5 in the Supplementary Materials. Due to an oversight by the authors, some sub-figures were partially duplicated. The corrected Figures S2–S5 appear below. The authors state that the scientific conclusions are unaffected. This correction was approved by the Academic Editor. The original publication has also been updated.

**Figure S2:**
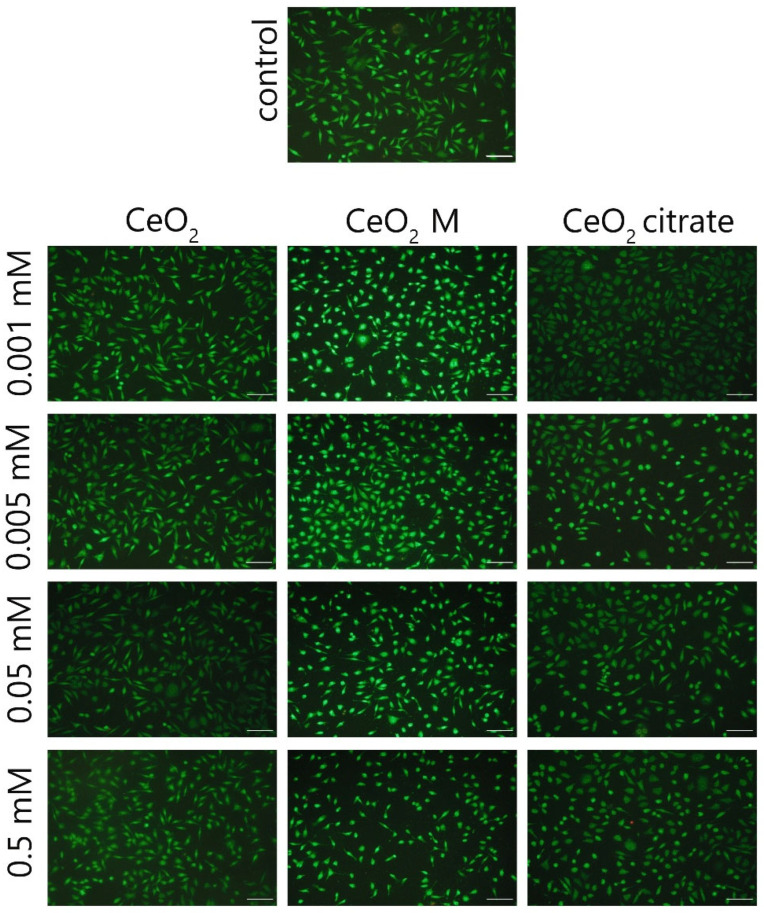
Microimages of the NCTC cell line L929 after staining with a mixture of dyes SYTO 9 (green)/propidium iodide (red) after 24 h of cultivation with bare CeO_2_ NPs, including those containing maltodextrin or ammonium citrate. The concentration is expressed in mg/mL. Scale bar is 100 microns.

**Figure S3:**
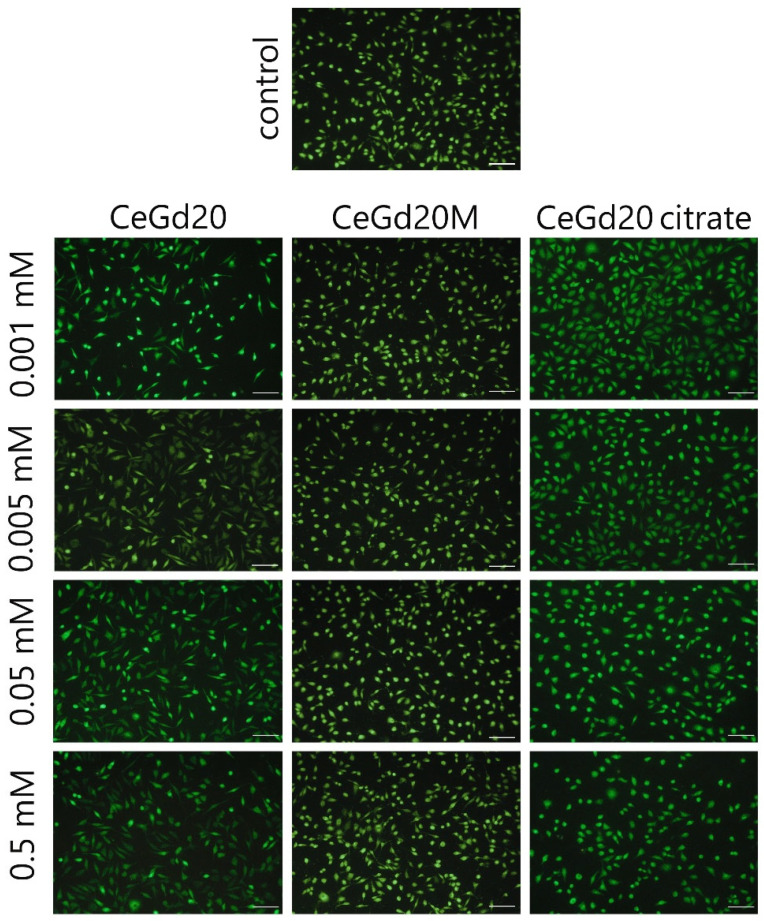
Microimages of the NCTC cell line L929 after staining with a mixture of dyes SYTO 9 (green)/propidium iodide (red) after 24 h of cultivation with CeO_2_:Gd NPs (20%), including those stabilised with maltodextrin or ammonium citrate. The concentration is expressed in mg/mL. Scale bar is 100 microns.

**Figure S4:**
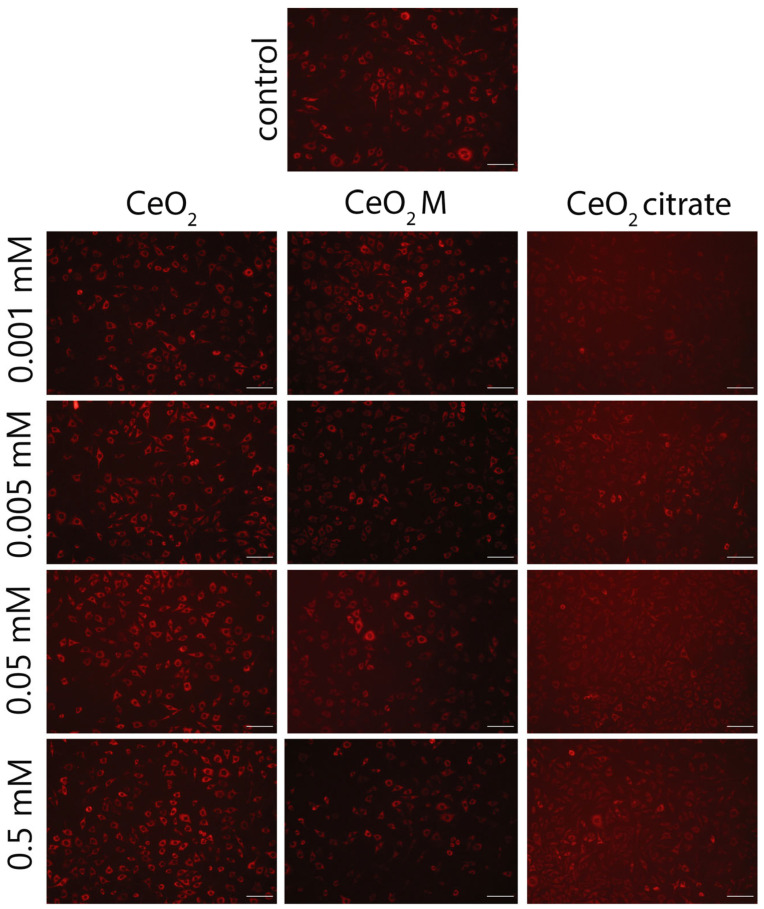
Microimages of the NCTC cell line L929 after staining with tetramethylrhodamine (TMRE) after 24 h of cultivation with bare CeO_2_ NPs, including those stabilised with maltodextrin or ammonium citrate. Scale bar is 100 microns.

**Figure S5:**
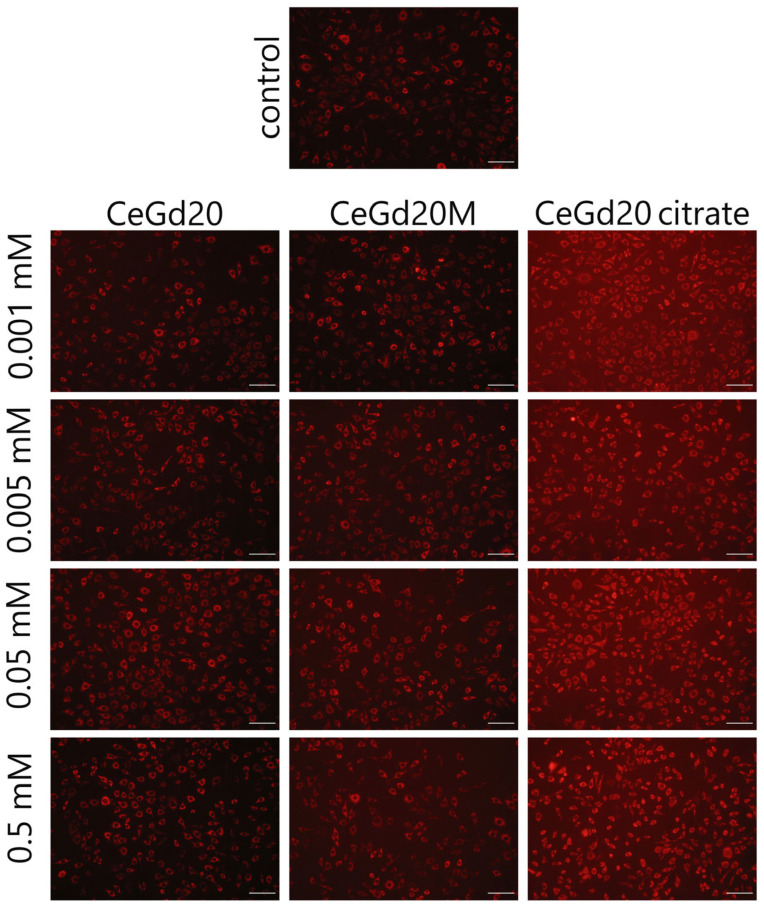
Microimages of the NCTC cell line L929 after staining with tetramethylrhodamine (TMRE) after 24 h of cultivation with CeO_2_:Gd NPs (20%), including those stabilised with maltodextrin or ammonium citrate. Scale bar is 100 microns.
