# Supplementary material for: Gadolinium Doping Modulates the Enzyme-like Activity and Radical-Scavenging Properties of CeO2 Nanoparticles
Source: Nanomaterials (Basel). 2024 Apr 26;14(9):769. doi: 10.3390/nano14090769 (PMC11085435; doi:10.3390/nano14090769)
Supplement: Supplementary file 1 [file nanomaterials-14-00769-s001.zip › nanomaterials-2953201-supplementary-update.pdf]

nanomaterials

MDPI

Electronic Supporting Information

Gadolinium Doping Modulates the Enzyme-like Activity and Radical-Scavenging Properties of CeO_2 Nanoparticles

Madina M. Sozarukova [1], Taisiya O. Kozlova [1], Tatiana S. Beshkareva [1,2], Anton L. Popov [3], Danil D. Kolmanovich [3], Darya A. Vinnik [3], Olga S. Ivanova [4], Alexey V. Lukashin [2], Alexander E. Baranchikov [1] and Vladimir K. Ivanov [1,*]

[1] Kurnakov Institute of General and Inorganic Chemistry of the Russian Academy of Sciences, 119991 Moscow, Russia
[2] Materials Science Department, Lomonosov Moscow State University, 119234 Moscow, Russia
[3] Institute of Theoretical and Experimental Biophysics of the Russian Academy of Sciences, 142290 Pushchino, Russia
[4] Frumkin Institute of Physical Chemistry and Electrochemistry of the Russian Academy of Sciences, 119071 Moscow, Russia
* Correspondence: van@igic.ras.ru

Analysis of the biological activity of CeO_2 NPs and gadolinium-doped CeO_2 NPs

S1. Materials and Methods

S1.1. Cell culture

The NCTC L929 cell line was used for cytotoxicity analysis. Cells were seeded onto a 96-well plate at a concentration of $15x10^3/cm^2$ in a DMEM/F12 cell medium (1:1), with 10% fetal bovine serum (FBS) and 100 U/ml penicillin/streptomycin and 2 mM L-glutamine, and cultured at 37°C in an atmosphere of 5% CO_2. After 16 hours, the culture medium was replaced with a medium containing a different concentration of gadolinium-doped CeO_2 NPs.

S1.2. MTT assay

Cell viability was assessed using MTT assay based on the reduction of a colourless tetrazolium salt (3-[4,5-dimethylthiazole-2-yl]-2,5-diphenyl tetrazolium bromide, MTT). After 24 hours of culturing, 0.5 mg/mL MTT reagent was added to the wells. The optical density of the formazan formed was measured at $\lambda = 540$ nm, using a BioRad 680 photometer.

S1.3. Live/Dead assay

An L-7007 LIVE/DEAD BacLight Bacterial Viability Kit (Invitrogen) was used to assess the ratio of living/dead cells in cell culture. This kit contains SYTO 9 dye (stains all cells, $\lambda = 485/498$ nm) and propidium iodide dye (stains the nuclei of dead cells, $\lambda = 535/617$ nm). Cell staining was performed by replacing the culture medium with a mixture of dyes (5 µM). Observation of morphology and fluorescent staining was carried out on a Zoe inverted microscope (BioRad, USA). Stained cells were counted using Image J software.

S1.4. Mitochondrial membrane potential (MMP level) analysis

The MMP level was assessed using tetramethyl rhodamine, TMRE (Thermo Fisher Scientific, USA). TMRE is a positively charged red-orange dye that penetrates cells and accumulates readily in active mitochondria, due to their negative membrane potential. Depolarised or inactive mitochondria have a reduced membrane potential and are unable to accumulate TMRE. The TMRE excitation peak was at 488 nm, the emission peak was at 575 nm. The working solution containing the dye was prepared in Hanks solution. After

the addition of the prepared solution to the cells, cultivation was carried out for 15 min. A Zoe inverted fluorescence microscope (BioRad, USA) was used, after which the level of fluorescence intensity was calculated using ImageJ software.

S2. Results

S2.1. Study of the dehydrogenase activity level

The metabolic activity analysis for the NCTC L929 line cell culture of mouse fibroblasts showed that, after incubation with nanoparticles synthesised without a stabiliser (Fig. S1), nanoparticles of the undoped ceria sol reliably increased (*p = 0.0001***) the metabolic activity of mouse fibroblasts. After 24 hours of incubation in maximum concentration (0.5 mg/ml) CeO_2:Gd NPs (20%) exhibited a more pronounced dose-dependent stimulation effect for this type of cell, significantly increasing the optical density value for concentrations of 0.05 mg/ml and 0.5 mg/ml. An analysis of ceria sols (Fig. S1) containing stabilisers also revealed no significant decrease in the viability of NCTC L929 cells after the co-incubation. Only ceria sol stabilised with maltodextrin showed a slight decrease in the level of metabolic activity of mouse fibroblasts of the NCTC L929 line (*p = 0.0102) at the maximum concentration (500 μg/ml).

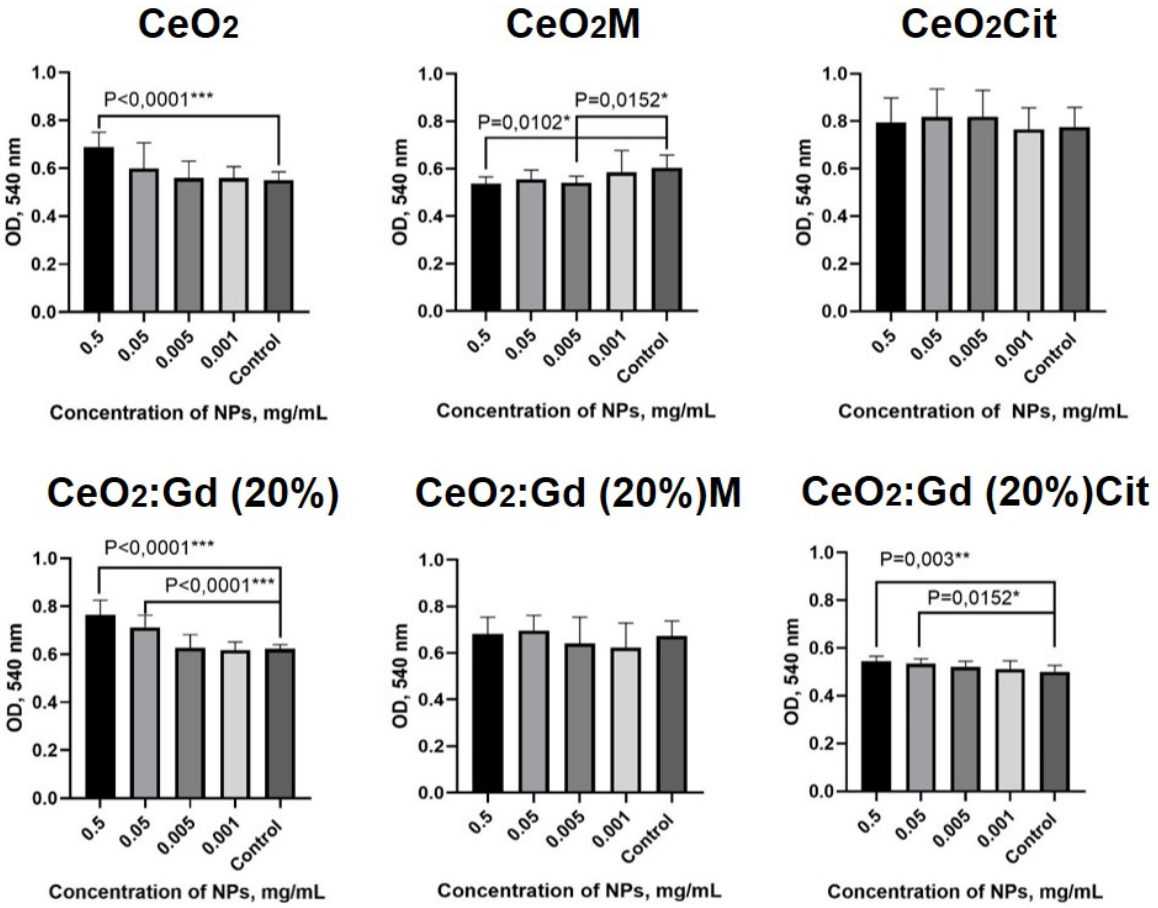

Figure S1. Assessment of the dehydrogenase activity level of NCTC L929 cells after 24 hours of cultivation with bare CeO_2 NPs or CeO_2:Gd NPs (20%), including those stabilised with maltodextrin or ammonium citrate. Data are presented as M±SD. The reliability of the results was calculated using the Mann-Whitney U test at p<0.05.

S2.2. Investigation of differential fluorescent staining (Live/Dead assay)

An analysis of the ratio of living and dead cells (Live/Dead assay) was carried out after incubation with cerium dioxide sols, including those containing maltodextrin or

ammonium citrate, in a wide concentration range (0.001–0.5 mg/ml). Fig. S2 confirms that the cells preserved the morphology characteristic of actively proliferating cells. The total number of cells is comparable to the untreated control group, which indirectly confirms the absence of a negative influence of nanoparticles on the mitotic function of cells.

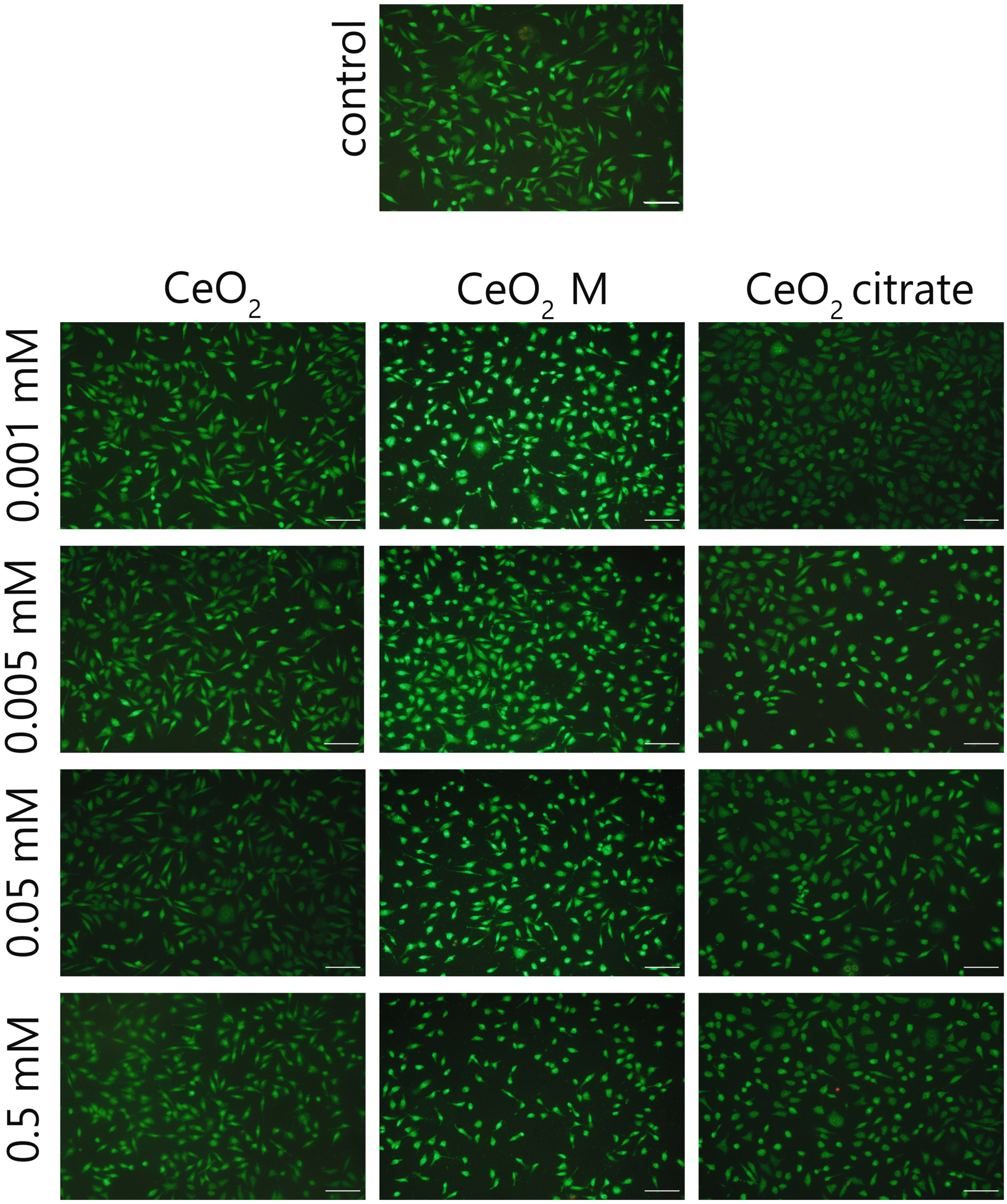

Figure S2. Microimages of the NCTC cell line L929 after staining with a mixture of dyes SYTO 9 (green)/propidium iodide (red) after 24 h of cultivation with bare CeO_2 NPs, including those containing maltodextrin or ammonium citrate. The concentration is expressed in mg/mL. Scale bar is 100 microns.

Analogous results were obtained when analysing microimages of NCTC L929 cell culture after their incubation with CeO_2:Gd NPs (20%), including those stabilised with maltodextrin or ammonium citrate (Fig. S3). The microimages confirm the absence of cells positively stained with propidium iodide and the characteristic morphology of fibroblasts (expansion, degree of cytoplasmic granulation and microtopology).

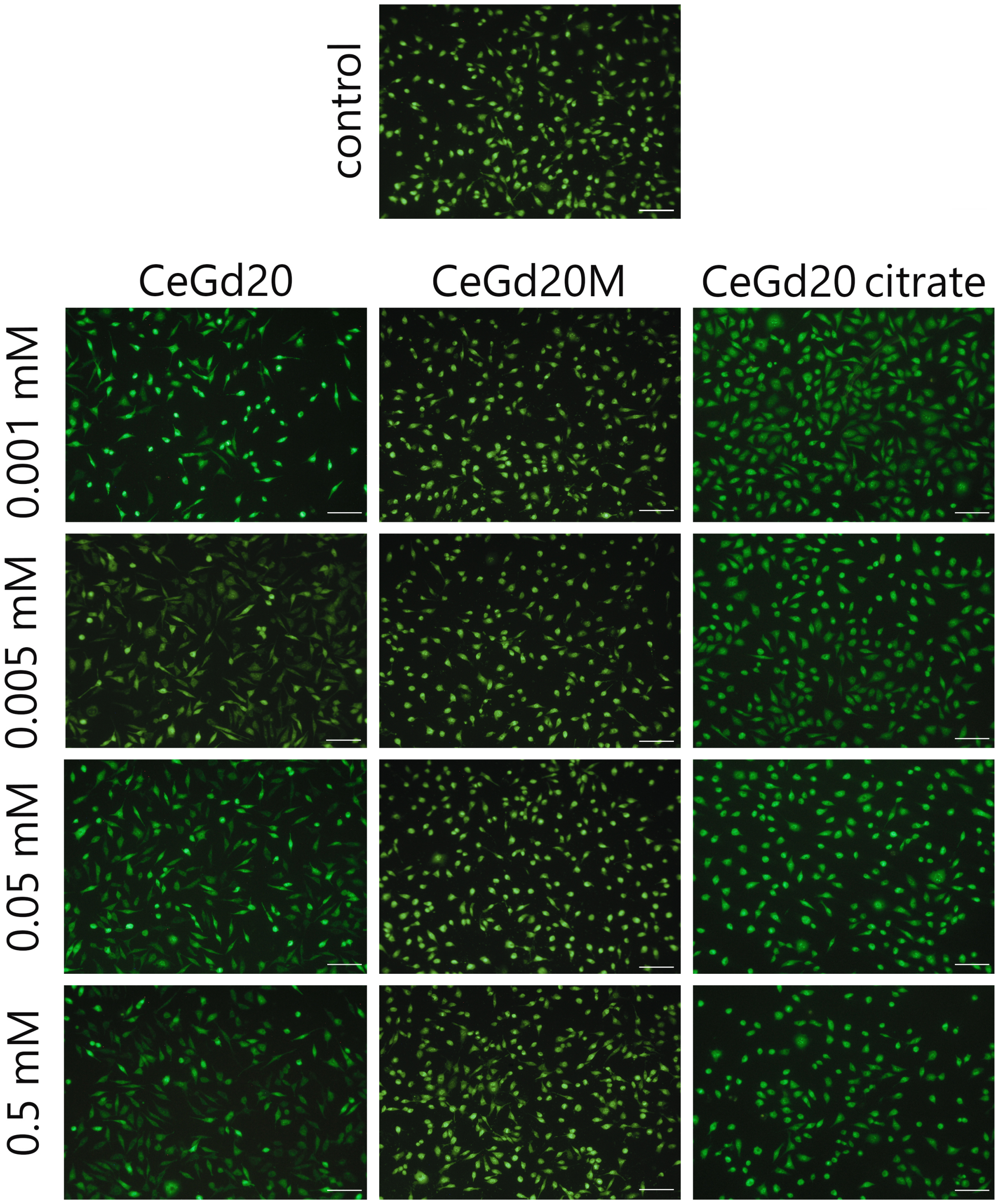

Figure S3. Microimages of the NCTC cell line L929 after staining with a mixture of dyes SYTO 9 (green)/propidium iodide (red) after 24 h of cultivation with CeO_2:Gd NPs (20%), including those stabilised with maltodextrin or ammonium citrate. The concentration is expressed in mg/mL. Scale bar is 100 microns.

S2.3. Investigation of the effect of nanoparticles on the mitochondrial membrane potential of cells

The violation of the electron-transport chain function of mitochondria includes changes in their membrane potential (MMP). It was previously shown that cerium-containing nanoparticles can depolarise the mitochondrial membrane of human colon cancer cells, causing their death *via* the mitochondrial pathway of apoptosis.[1] Thus, an analysis was made of the level of the mitochondrial membrane potential of cell culture mouse fibroblasts after incubation with ceria sols at different concentrations. It was revealed that the 24-hour incubation of cells of the NCTC L929 line, with all investigated samples (Fig. S4, S5), in a wide range of concentrations (0.001–0.5 mg/ml), did not lead to any changes in MMP, which confirms their high biocompatibility.

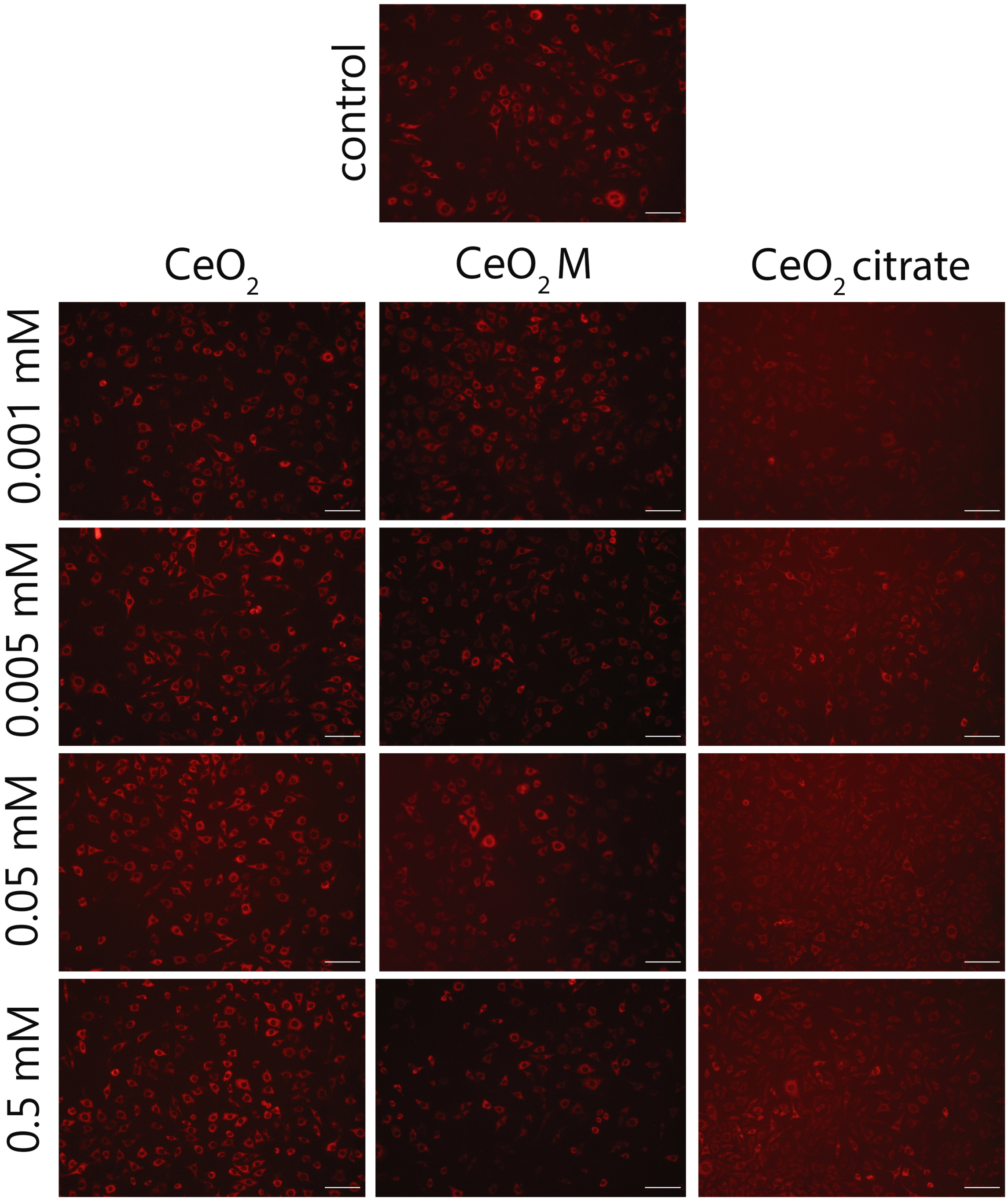

Figure S4. Microimages of the NCTC cell line L929 after staining with tetramethylrhodamine (TMRE) after 24 h of cultivation with bare CeO_2 NPs, including those stabilised with maltodex-trin or ammonium citrate. Scale bar is 100 microns.

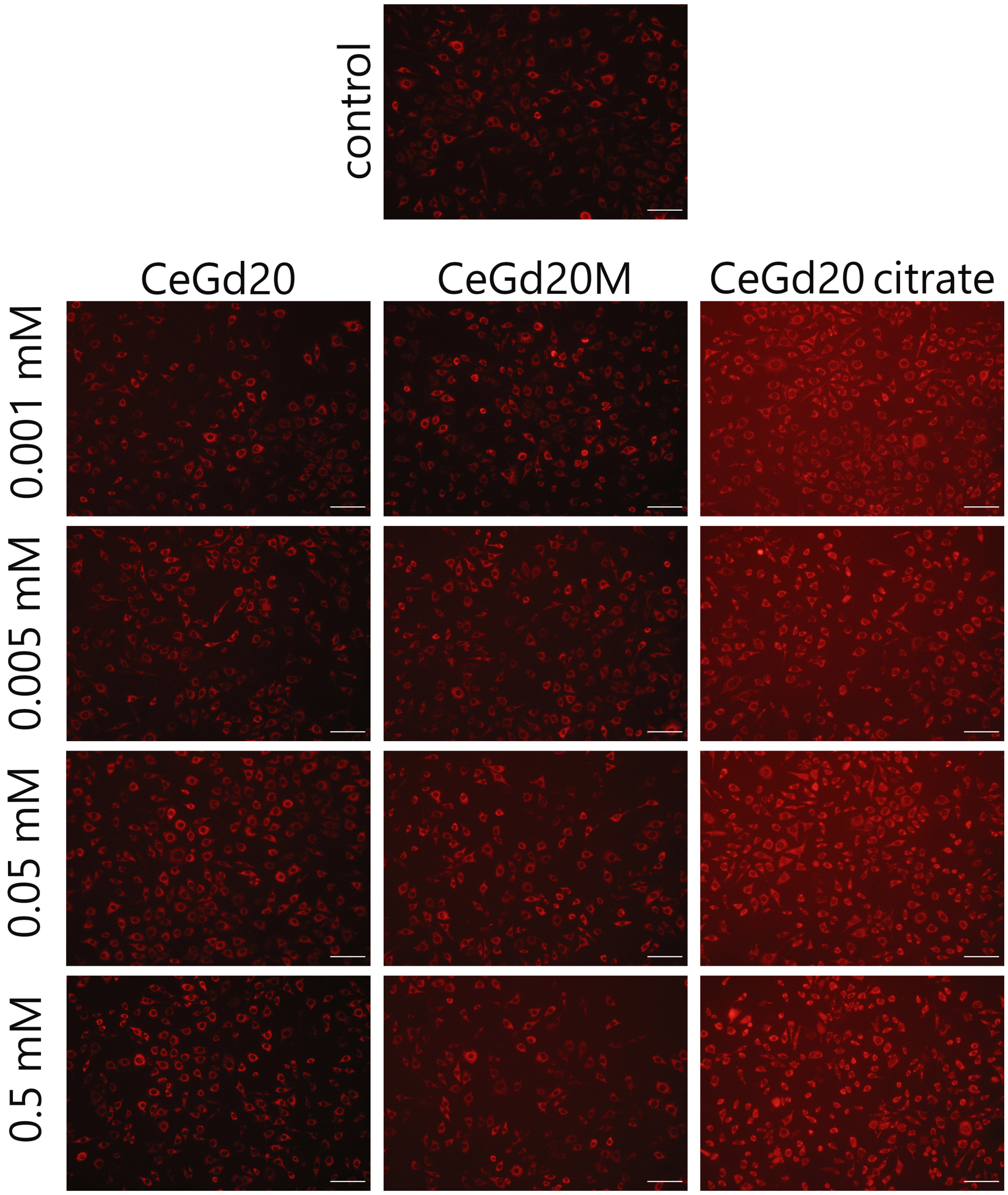

Figure S5. Microimages of the NCTC cell line L929 after staining with tetramethylrhodamine (TMRE) after 24 h of cultivation with CeO_2:Gd NPs (20%), including those stabilised with maltodextrin or ammonium citrate. Scale bar is 100 microns.

References

1. Datta, A.; Mishra, S.; Manna, K.; Saha, K. D.; Mukherjee, S.; Roy, S., Pro-oxidant therapeutic activities of cerium oxide nanoparticles in colorectal carcinoma cells. *ACS omega* **2020,** *5* (17), 9714-9723.

Disclaimer/Publisher's Note: The statements, opinions and data contained in all publications are solely those of the individual author(s) and contributor(s) and not of MDPI and/or the editor(s). MDPI and/or the editor(s) disclaim responsibility for any injury to people or property resulting from any ideas, methods, instructions or products referred to in the content.
